# The prevention and improvement effects of vitamin D on type 2 diabetes mellitus: evidence from an umbrella review on Meta-analyses of cohort studies and randomized controlled trials

**DOI:** 10.3389/fnut.2024.1462535

**Published:** 2024-10-25

**Authors:** Le Cheng, Chenhui Lv, Lushan Xue, Cheng Zhang, Lili Wang, Xi Wang, Shuangzhi Chen, Xuemin Li, Wenjuan Feng, Haoran Xie, Haifeng Zhao

**Affiliations:** ^1^Nutritional and Food Science Research Institute, Department of Nutrition and Food Hygiene, School of Public Health and Center for Ecological Public Health Security of Yellow River Basin, Shanxi Medical University, Taiyuan, Shanxi, China; ^2^Department of Clinical Nutrition, Shanxi Bethune Hospital, Shanxi Academy of Medical Science, Tongji Shanxi Hospital, Third Hospital of Shanxi Medical University, Taiyuan, Shanxi, China; ^3^Center for Disease Control and Prevention in Shanxi Province, Taiyuan, Shanxi, China; ^4^MOE Key Laboratory of Coal Environmental Pathogenicity and Prevention, Ministry of Education, Taiyuan, China

**Keywords:** vitamin D, type 2 diabetes mellitus, Meta-analyses, cohort studies, randomized controlled trials, umbrella review

## Abstract

**Background:**

To clarify whether Vitamin D prevent the occurrence of type 2 diabetes mellitus (T2DM) and improve glucose control in T2DM patients, we conducted this umbrella review, taking into account the inconsistent results of existing Meta-analyses. We aim to reveal the causal relationship between Vitamin D and T2DM through summarizing Meta-analyses of observational studies, and clarify the improvement on glucose control in T2DM patients through summarizing Meta-analyses of RCT studies between Vitamin D supplementation and T2DM patients, especially in T2DM patients with Vitamin D deficiency.

**Methods:**

We collected the Meta-analyses of observational studies and RCTs in PubMed, Scopus, Embase, Web of Science, and Cochrane.

**Results:**

16 Meta-analyses (6 effect sizes for cohort studies and 10 effect sizes for RCTs) were included in the umbrella Meta-analyses. Random-effects model was carried out to calculate the pooled point estimates and their respective 95% confidence intervals (CI). The results revealed that lower 25(OH)D levels increased the risk of T2DM (Pooled ES_RR_ = 1.34; 95%CI: 1.16, 1.53), Vitamin D supplementation ameliorated FBG (ES = −0.56; 95%CI: −1.00, −0.11), HbA1c (ES = −0.11; 95%CI: −0.20, −0.02), insulin (ES = −0.38; 95%CI: −0.59, −0.18) and HOMA-IR (ES = −0.37; 95%CI: −0.57, −0.16) in T2DM patients, especially in those with Vitamin D deficiency (FBG = −0.98; HbA1c = −0.27; HOMA-IR = −0.52).

**Conclusion:**

The present umbrella Meta-analyses demonstrates the potential benefits of higher serum Vitamin D levels and Vitamin D supplementation in reducing the development and symptoms of T2DM.

## Graphical abstract

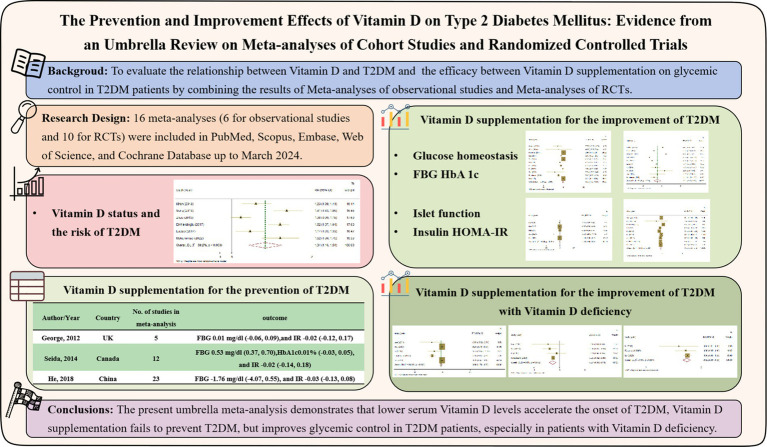

## 1 Introduction

Type 2 diabetes mellitus (T2DM) is a multifactorial disease involving lifestyle and nutritional status, characterized by the impairment of insulin secretion, insulin resistance (IR), or the combination of both, bringing about persistent inflammation and hyperglycemia (1). According to the data of IDF global diabetes survey (10th Edition), the number of adults aged 20–79 with diabetes in the world reached 537 million in 2021, in which the prevalence rate of the elderly aged 75–79 reached 24.0%, which has become the global public health issue of concern (2). Meanwhile, due to the increased prevalence and prolonged duration of T2DM, complications related to T2DM are also highly prevalent (3). T2DM patients reduce their intake of foods with higher Vitamin D concentration, such as animal liver and egg yolks, due to the control of energy and lipid intake. This makes them become more prone to Vitamin D deficiency. In numerous studies investigating the risk factors of T2DM, they have been found that the baseline Vitamin D status is closely related to T2DM. The Meta-analyses of 28 prospective studies on plasma 25(OH)D_3_ status and T2DM risk showed that comparing with the participants in the lowest category (< 10 ng/mL, severe deficiency), the pooled RR with 20–30 ng/mL 25(OH)D_3_ levels (insufficient) was 0.77 (95% CI = 0.72–0.82), with the highest 25(OH)D_3_ levels (> 30 ng/mL, adequate) was 0.66 (95% CI = 0.61–0.73) (4).

In the background of the development of T2DM, numerous molecular mechanisms support the role of Vitamin D on glycemic control (5–7). Pancreatic *β*-cells contain all elements which convert inactive Vitamin D into active metabolites, which is also the necessary process for insulin secretion (5, 6). In addition, activated Vitamin D further affect the expression of genes involving in insulin signal transduction (7). So, lower serum Vitamin D levels have been hypothesized to enhance IR and accelerate the development of diabetes, through affecting on islet cell function and survival (8, 9). Hence, the notion that Vitamin D deficiency may increase the risk of T2DM is physiologically feasible. In the past decade, many Meta-analyses of observational studies and randomized controlled trials (RCT) related to Vitamin D and T2DM have been published. Wherein, some of them have shown that supplementation Vitamin D will improve glucose metabolism in T2DM patients, but others reported null results (10–19). Therefore, considering the conflicting results among existing studies and the inability to draw definitive conclusions from these studies, we conducted this umbrella review. Umbrella review is an effective method for the systematic evaluation of data from multiple places and be useful in identifying potential biases within exposure-outcome connections.

This umbrella review aimed to clarify the protective role of Vitamin D on T2DM from onset to development. To our knowledge, no previous review has been made to summarize and appraise evidence obtained in Meta-analyses of cohort studies and RCTs on the prevention and improvement effects of Vitamin D and T2DM. This study is the first umbrella review to summarize existing evidences. Hence, we planned to summarize the Meta-analyses of observational studies on the relationship between Vitamin D and the risk of T2DM, to demonstrate the effect of lower levels of Vitamin D on the onset of T2DM. Subsequently, we proposed to pool Meta-analyses of RCTs on the prevention of T2DM with Vitamin D supplementation, as well as on the improvement of T2DM after Vitamin D supplementation. On this basis, we further analyzed the improvement of Vitamin D supplementation in T2DM patients with Vitamin D deficiency. We aim to clarify the key role of Vitamin D on T2DM onset and development.

## 2 Materials and methods

The review procedure has been registered in the International Prospective Register of Systematic Reviews (PROSPERO; Registration ID: CRD42024508755).^1^ We performed to the Preferred Reporting Items for the Systematic Reviews and Meta-analyses (PRISMA) statement guidelines (20).

### 2.1 Search strategy and study selection

We conducted an electronic search of Meta-analyses published up to March 20th, 2024, and searched the database of PubMed, Scopus, Embase, Web of Science, and Cochrane Database. We also searched PROSPERO. Based on the keywords (Vitamin D, cholecalciferol, and ergocalciferol) AND (type 2 diabetes mellitus, type 2 diabetes and T2DM) OR (fasting blood glucose, FBG, glycosylated hemoglobin, type A1C, HbA1c, insulin, homeostatic model assessment of insulin resistance, HOMA-IR, insulin resistance, glycemic control) AND (Meta-analyses), a structured search strategy was determined. Only publications written in English were included. To enhance the sensitivity of the search results, the wild-card phrase “*” was used. The reference lists from included studies were manually screened to ensure that all articles were contained.

### 2.2 Inclusion and exclusion criteria

For the current umbrella review, we included Meta-analyses of observational studies (cross-sectional, case–control and cohort) and RCTs considering the following criteria: reported effect sizes (ESs) and their corresponding confidence intervals (CI) for the effect of Vitamin D supplementation on glucose homeostasis (FBG and HbA1c) and islet function (insulin and HOMA-IR). Other studies were excluded, including original experimental studies, case reports, *in vitro*, *ex-vivo*, and *in vivo* investigations.

### 2.3 Data extraction

The articles were checked by two independent reviewers (LC and LX) to make sure they met with the eligibility criteria. The reviewers initially screened over the articles based on the abstracts and titles. Subsequently, they assessed the entire texts and determined their appropriateness for the Meta-analyses. Any disagreement was resolved with the third reviewer (HZ) through consensus.

The following data were extracted from the observational studies including cross-sectional, case–control and cohort: author, publication year, country, study design, sample size, age range, Vitamin D status and follow-up duration in observational studies, and the metrics of risk ratio (RR) or odds ratio (OR) and their 95% CI, outcome, publication bias and quality.

The following data were extracted from the RCTs studies: author, publication year, country, study design, sample size, age range, the dosage and duration of the intervention of Vitamin D supplementation and the metrics of weighted mean difference (WMD), standard mean difference (SMD) or hazard ratio (HR) and their 95% CI, outcome, publication bias and quality. For articles lacking necessary information, we asked the author via email to obtain this information.

### 2.4 Quality assessment

The assessment of multiple systematic reviews (AMSTAR2) questionnaire, as a methodological quality evaluation tool for systematic review/Meta-analysis, was used to assess the methodological quality of the qualifying studies by two reviewers (LC and CZ) independently. The questionnaire includes 16 items that ask reviewers to reply “Yes” or “Partial Yes” or “No,” and be categorized into “critically low quality,” “low quality,” “moderate quality,” and “high quality” (21). Also the third reviewer (HZ) resolved any disagreements. We evaluated the overall certainty of the evidence by Grading of Recommendations, Assess, Development and Evaluation (GRADE) tools. The quality of evidence was classified into four categories, “high,” “moderate,” “low,” and “very low” (22).

### 2.5 Data synthesis and statistical analysis

Using the random effects model, the overall effect sizes were computed via combining the point estimates and corresponding 95% CIs for observational and RCT studies. Cochrane’s Q test and the I^2^ index were utilized to discover statistical heterogeneity. The *p*-value for the Q-test of less than 0.1 or the I^2^ value greater than 40% were regarded as significant between-study heterogeneity. To investigate the impact of each study on the pooled point estimate, we performed the sensitivity analysis in which each of the studies were omitted. The Egger’s tests were used to explore the small-study effect, and the visual assessment of funnel plots were performed to assess publication bias. The version 16.0 of STATA was used to conduct all statistical analyses (Stata Corporation, College Station, TX). *p* < 0.05 was considered as significant.

## 3 Results

### 3.1 Study selection

**Figure 1:**
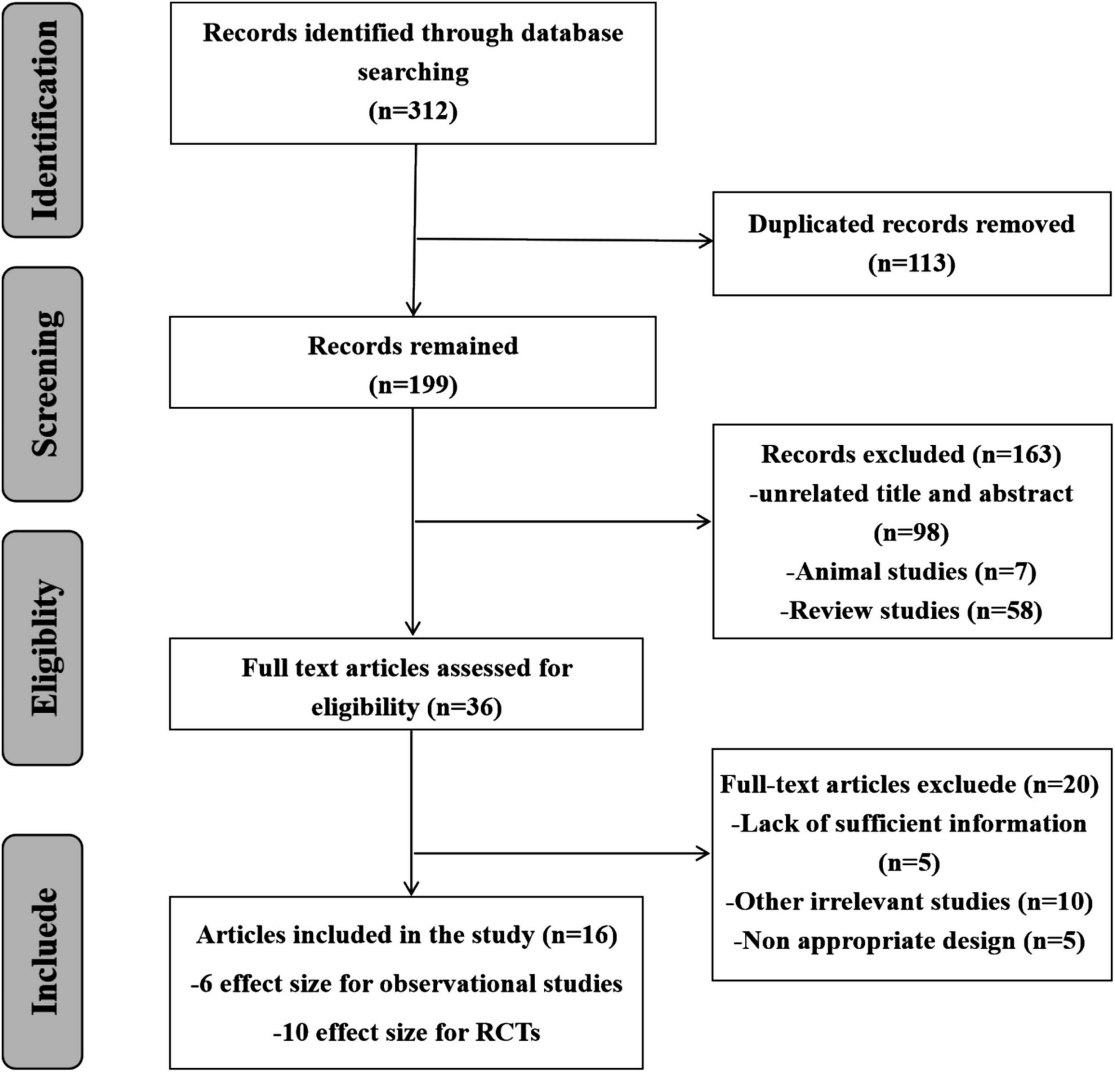
PRISMA flow diagram for Meta-analyses of observational studies and RCT.

Following the thorough search of electronic databases, 312 records were included. 113 papers were removed by reason of duplication, 163 studies were rejected due to inappropriate titles, abstracts and irrelevant studies. After reviewing 36 full texts, 20 studies were eliminated for useless and lacking necessary information. Finally, 16 Meta-analyses (6 effect sizes for cohort studies and 10 effect sizes for RCTs) were included. Figure 1 schematically showed the study selection process in the PRISMA study flow chart.

### 3.2 Lower serum vitamin D levels accelerate the onset of T2DM

We summarized the Meta-analyses of cohort studies to clarify the association between Vitamin D levels and the onset of T2DM, six Meta-analyses were included. The qualified articles were published between 2013 and 2023. The number of subjects ranged between 28,258 and 190,626. The average age of participants ranged between 20 and 79 years. The follow-up duration was between 1.3 and 22 years. The studies were conducted in China, Austria, Italy, United Kingdom, Iran and United States (Table 1).

**Table 1:** Characteristics of included Meta-analyses of cohort studies.

Author/Year	Country	Study design	No. of studies in Meta-analyses	N (case)	Age range	Vitamin D status	Follow-up time	Outcome	Quality
Khan, 2013 (30)	UK	Cohort	14	190,626 (9399)	25–79	Vitamin D intake	3–22 y	the lowest V.S. the highest RR = 1.23 (1.09, 1.41)	NR
Song, 2013 (31)	USA	Cohort	21	76,220 (4996)	30–79	25(OH)D level	1.3–22 y	the lowest V.S. the highest RR = 1.61 (1.43, 1.85)	NR
Zhao, 2013 (32)	China	Cohort	4	187,592 (9456)	NR	Vitamin D intake	5–20 y	the lowest V.S. the highest RR = 1.08 (0.99, 1.18)	NR
Ekmekcioglu, 2017 (4)	Austria	Cohort	28	133,657	NR	25(OH)D level	NR	the lowest V.S. the highest RR = 1.52 (1.37, 1.64)	NR
Lucato, 2017 (33)	Italy	Cohort	9	28,258 (2863)	47–76.5	25(OH)D level: 46.1–80.1 nmol/L	2–11 y	the lowest V.S. the highest RR = 1.17 (1.03, 1.33)	NOS
Mohammadi, 2022 (34)	Iran	Cohort	17	127,980	30–75	25(OH)D status	3–22 y	the lowest V.S. the highest RR = 1.52 (1.30, 1.79)	NOS

*Outcome: The lowest V.S. the highest of RR was extracted from the data reported in the corresponding literature. For those studies that reported RR for the highest V.S. the lowest, we inverted RR and its lower and upper limits to compute the estimate for the lowest V.S. the highest. Abbreviations: NOS, the Newcastle-Ottawa Scale; NR, not reported; RR, risk ratio.

**Figure 2:**
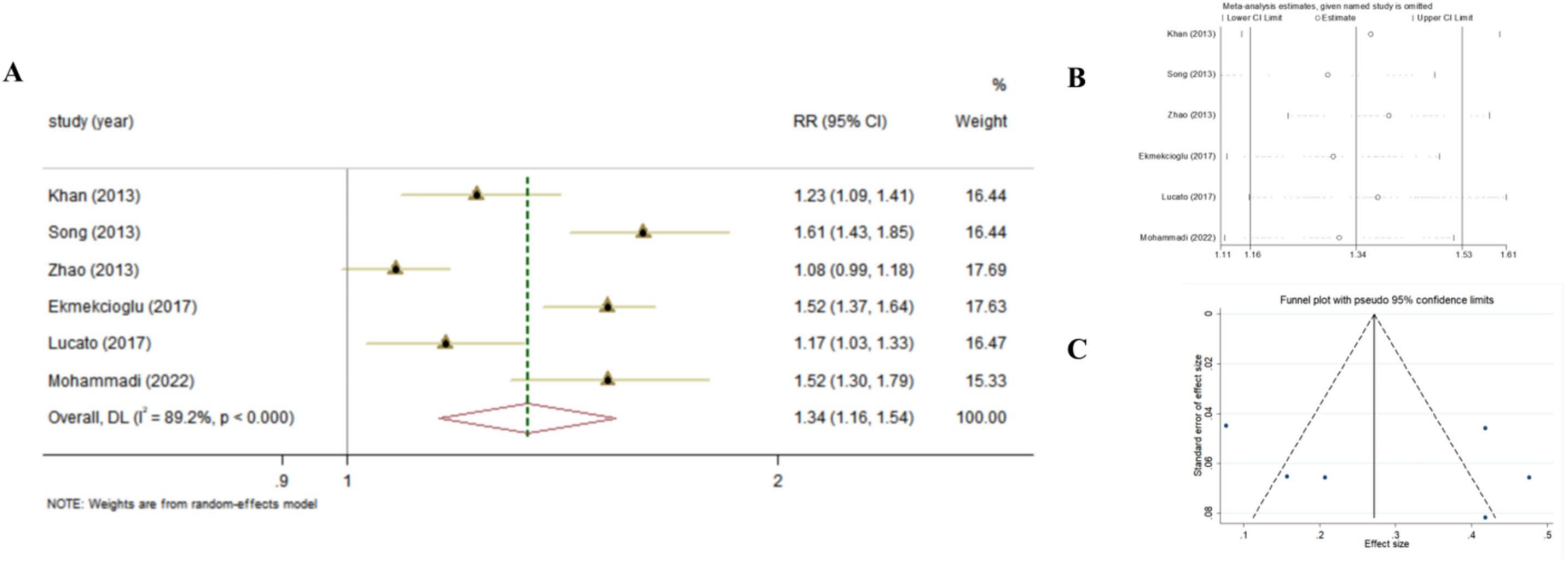
The effect of Vitamin D levels on the risk of T2DM according to Meta-analyses of cohort studies. **(A)** Forest plot; **(B)** Sensitivity analysis; **(C)** Funnel plot.

Combining the results using random-effects model, our findings revealed that compared to the highest serum levels of Vitamin D, the lowest serum levels of Vitamin D increased the risk of developing T2DM (Pooled ES_RR_ = 1.34; 95% CI: 1.16, 1.53, *p* < 0.001; Figure 2). The level of heterogeneity was high (I^2^ = 88.8%, *P*-heterogeneity <0.001). Subsequently, we removed each study separately, and the heterogeneity remained significant. Finally, we conducted a sensitivity analysis, the results showed that there was no significant change in ES and 95% CI, indicating that the results were relatively robust. No significant publication bias was observed with funnel plot and Egger’s test (*p* = 0.583).

### 3.3 Vitamin D supplementation fails to prevent T2DM

Subsequently, we summarized Meta-analyses of RCTs on the prevention of T2DM with Vitamin D supplementation, considering that Vitamin D deficiency promotes the onset of T2DM. There were three Meta-analyses (29 RCTs on the prevention of T2DM with Vitamin D supplementation in non-T2DM population, excluding studies that were dually included) reported on glycemic control. In this summary analysis, there were relatively few studies that was considered eligible, so the systematic review was conducted. According to the 3 studies included, we found that Vitamin D supplementation did not affect the FBG (ES = 0.01, 95%CI: −0.06, 0.09; ES = 0.53, 95%CI: 0.37, 0.70; ES = −1.76, 95%CI: −4.07, 0.55) and HOMA-IR (ES = −0.02, 95%CI: −0.12, 0.17; ES = −0.02, 95%CI: −0.14, 0.18; ES = −0.03, 95%CI: −0.13, 0.08) for individuals with normal glucose tolerance. Only one study analyzed the HbA1c (ES = 0.01, 95%CI: −0.03, 0.05), but no significant effect was found (Table 2).

**Table 2:** Characteristics of included Meta-analyses of RCT studies.

Population	Author/Year	Country	No. of studies in Meta-analyses	No. of participants	Age range	Dosage	Duration time	Outcome	Quality
Non-T2DM	George, 2012 (10)	UK	5	1,038	43–71	400–8,571.4 IU/d 1 μg paricalcitol	6–144 w	FBG 0.01 mg/dL (−0.06, 0.09), and IR-0.02 (−0.12, 0.17)	NA
	Seida, 2014 (11)	Canada	12	1,978	23–71	125–8,571.4 IU/d	6–144 w	FBG 0.53 mg/dL (0.37, 0.70), HbA1c 0.01% (−0.03, 0.05), and IR-0.02 (−0.14, 0.18)	Cochrane 5/12 high
	He, 2018 (37)	China	23	NR	NR	NR	NR	FBG-1.76 mg/dL (−4.07, 0.55), and IR-0.03 (−0.13, 0.08)	JADAD 16/23 high
T2DM	George, 2012 (10)	UK	15	40,226	26–77	400–120,000 IU/d	1–336 w	FBG-5.76 mg/dL (−10.26, −1,26), HbA1c 0.03% (−0.18, 0.23), and IR-0.25 (−0.48, −0.03)	NA
	Seida, 2014 (11)	Canada	11	503	40–66	1,000–450,000 IU/d	4–48 w	FBG-4.86 mg/dL (−17.56, 7.84), HbA1c-0.20% (−0.52, 0.11), and IR-1.46 (−4.27, 1.34)	Cochrane 1/11 high
	Krul-Poel, 2017 (12)	Netherlands	23	1,797	44–67	400–300,000 IU/d	4–48 w	FBG 0.09 mg/dL (−0.11, 0.28), HbA1c 0.12% (−0.03, 0.26), and IR 0.23 (−0.06, 0.53)	Cochrane 14/23 high
	Lee, 2017 (13)	USA	22	T: 1,351\u00B0C: 1,336	48–70	400–300,000 IU/d	8–287 w	FBG-2.33 mg/dL (−6.62, 1.95), HbA1c-0.32% (−0.53, −0.01)	Cochrane 7/22 high
	Wu, 2017 (14)	China	25	T: 921\u00B0C: 953	49–67	400–300,000 IU/d	4–48 w	FBG-0.14 mg/dL (−0.31, 0.03), and HbA1c-0.25% (−0.45, −0.05)	JADAD 4/20 high
	Li, 2018 (15)	China	20	T: 1,244\u00B0C:1,459	47–73.27	20–300,000 IU/d	8–24 w	FBG-3.59 mg/dL (−7.94, 0.76), HbA1c-0.14% (−0.37, 0.08), insulin-0.84 pmol/L (−2.27, 0.06), and IR-0.57 (−1.09, −0.04)	Cochrane 4/20 high
	Hu, 2019 (16)	China	19	T: 747\u00B0C: 627	NR	1,000–300,000 IU/d	4–48 w	Short-term (2–6 m): FBG-2.52 mg/dL (−5.22, 0.18), HbA1c-0.16% (−0.27, −0.04), insulin-0.57 pmol/L (−0.78, −0.35), and IR-0.75 (−0.97, −0.53) Long-term (> 6 m): FBG-0.54 mg/dL (−4.32, 3.24), HbA1c 0.08% (−0.12, 0.29), insulin-0.25 pmol/L (−0.64, 0.14), and IR-0.26 (−0.60, 0.09)	JADAD 17/19 high
	Zou, 2021 (17)	China	15	T: 555\u00B0C: 546	42.4–66.1	2000–400,000 IU/d	2–26 w	FBG-8.74 mg/dL (−17.00, −0.48), HbA1c-0.11% (−0.32, 0.10), insulin-9.06 pmol/L (−29.90, 11.78), and IR-0.46 (−0.88, −0.04)	NR
	Farahmand, 2023 (18)	Iran	46	T: 2,164\u00B0C: 2,149	29.9–72.2	20–400,000 IU/d	4–48 w	FBG-5.02 mg/dL (−6.75, −3.28), HbA1c-0.20% (−0.29, −0.11), and IR-0.42 (−0.76, −0.07)	Cochrane 8/46 high
	Lei, 2023 (19)	China	18	T: 611\u00B0C: 632	38.5–73.3	20–100,000 IU/d	8–24 w	FBG-0.17 mg/dL (−0.301, −0.039), HbA1c-0.26% (−0.39, −0.14), and IR-0.441 (−0.58, −0.30)	Cochrane 6/18 high

*Outcome: The effect size of each indicator is extracted from the pooled effect size reported in the included Meta-analysis. For those reports with inconsistent units, we have made unit conversions. Abbreviations: C, control group; FBG, fasting blood glucose; HbA1c, glycosylated hemoglobin, type A1C; HOMA-IR, homeostatic model assessment of insulin resistance; JADAD, Jadad Scale; NA, not available; NR, not reported; T, treatment group.

### 3.4 Vitamin D supplementation improves glycemic control in T2DM patients

It seemed useless that Vitamin D supplementation ameliorates glycemic control in non-T2DM patients, we further summarized Meta-analyses of RCTs on the Vitamin D supplementation in T2DM patients. In this umbrella Meta-analyses, 10 studies were contained. These Meta-analyses were published from 2012 to 2023. The number of subjects ranged between 503 and 40,226. The average age of participants ranged between 26 and 77 years. Duration of intervention was between 1 and 336 weeks. Dosages of Vitamin D used varied between 20 and 450,000 IU/day. Studies were conducted in China, United States, Iran, United Kingdom, Canada and Netherlands (Table 2).

#### 3.4.1 Risk of bias assessment

**Figure 3:**
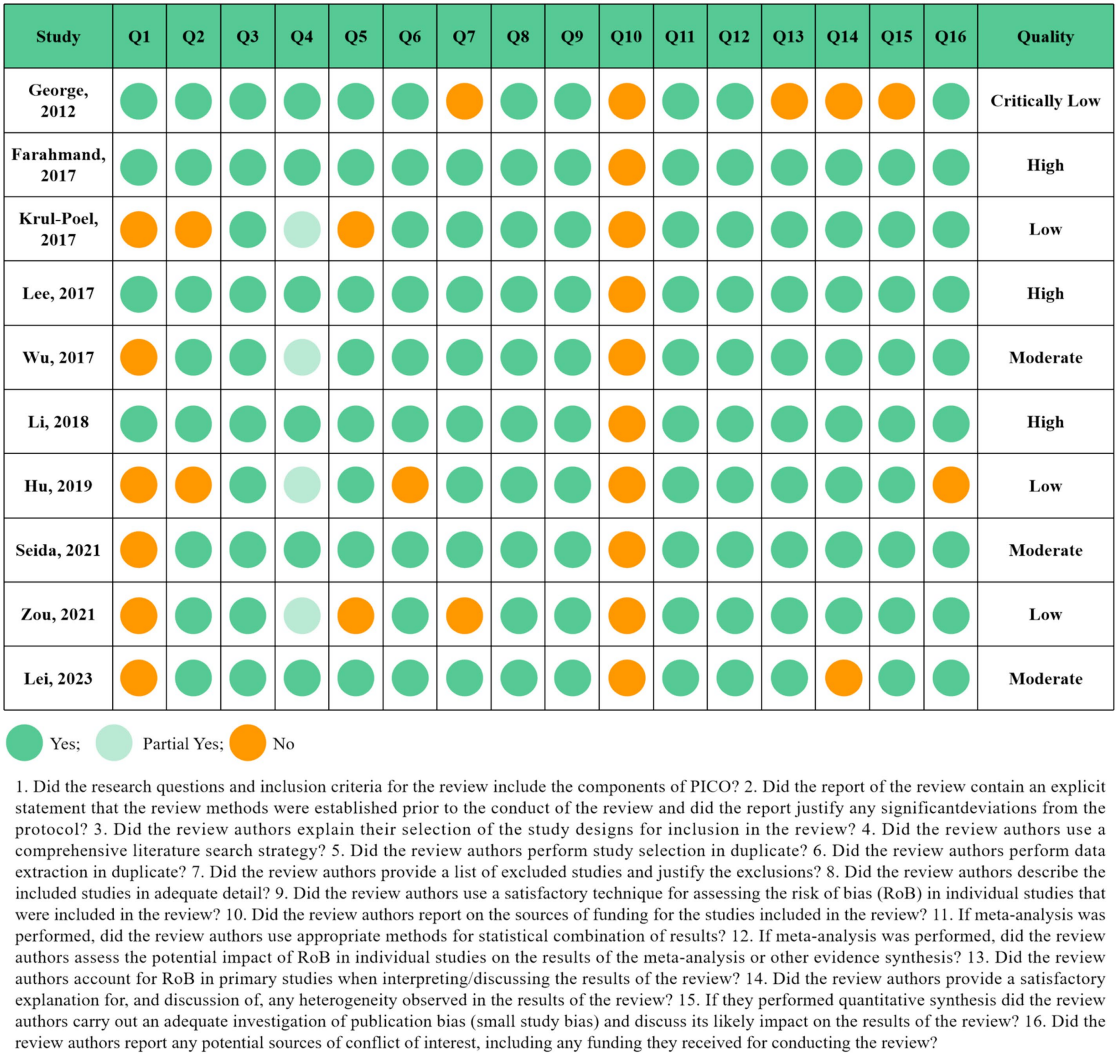
Results of assessment of the methodological quality of the Meta-analyses.

The results of the quality assessment according to the AMSTAR2 questionnaire were displayed in Figure 3. Out of 10 Meta-analyses of RCTs, four studies were evaluated as low and critically low-quality studies. Three in 10 studies were classified as moderate quality and three as high quality. Item 10 (Did the review authors report on the sources of funding for the studies included in the review?) was displayed as “No” in all included Meta-analyses. Although it appears as a non-critical domain in the AMSTAR2 questionnaire, it is still recommended to add this section in the following Meta-analyses.

#### 3.4.2 Vitamin D supplementation and FBG in T2DM patients

**Figure 4:**
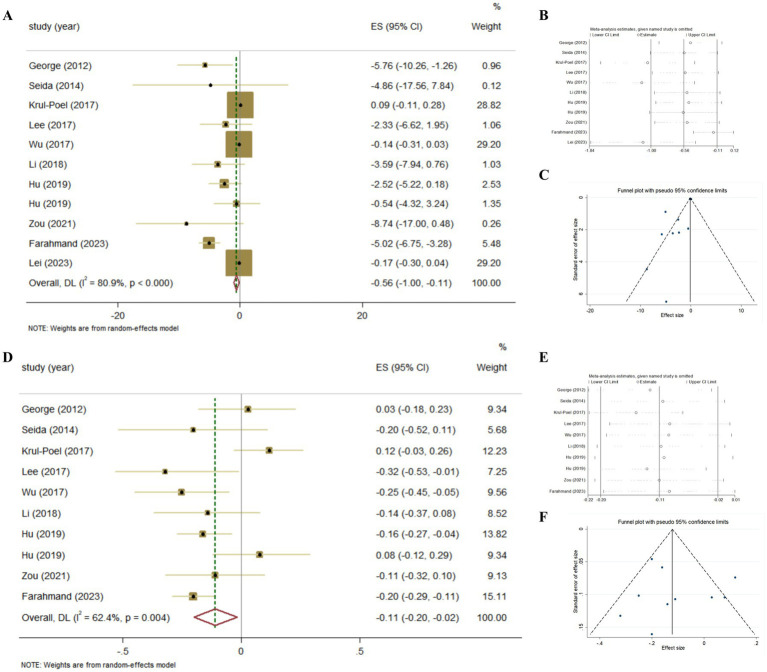
The effect of Vitamin D supplementation on glucose homeostasis in T2DM patients according to Meta-analyses of RCT. **(A)** Forest plot on FBG; **(B)** Sensitivity analysis on FBG; **(C)** Funnel plot on FBG; **(D)** Forest plot on HbA1c; **(E)** Sensitivity analysis on HbA1c; **(F)** Funnel plot on HbA1c.

The association between Vitamin D supplementation and the level of FBG was examined in all Meta-analyses. Combining the findings using random-effects model, the results revealed the significant effect (Pooled ES = −0.56; 95% CI: −1.00, −0.11, *p* = 0.015; Figures 4A–C), after converting the units of FBG to mg/dl. The level of heterogeneity was high (I^2^ = 80.9%, *P*-heterogeneity <0.001). Subsequently, we removed each study separately, and we found that the main source of heterogeneity was Farahmand, 2023. After removing this Meta-analyses, the heterogeneity was lower, but still significant (I^2^ = 58.2%, *P*-heterogeneity = 0.01), and the pooled ES showed no significant association between Vitamin D supplementation and FBG (Pooled ES = −0.16; 95% CI: −0.43, 0.12, *p* = 0.262). Then, we conducted the sensitivity analysis and the results also showed that Farahmand, 2023 was the main source of heterogeneity. The significant publication bias was observed in funnel plots and Egger’s test (*p* = 0.012).

**Figure 5:**
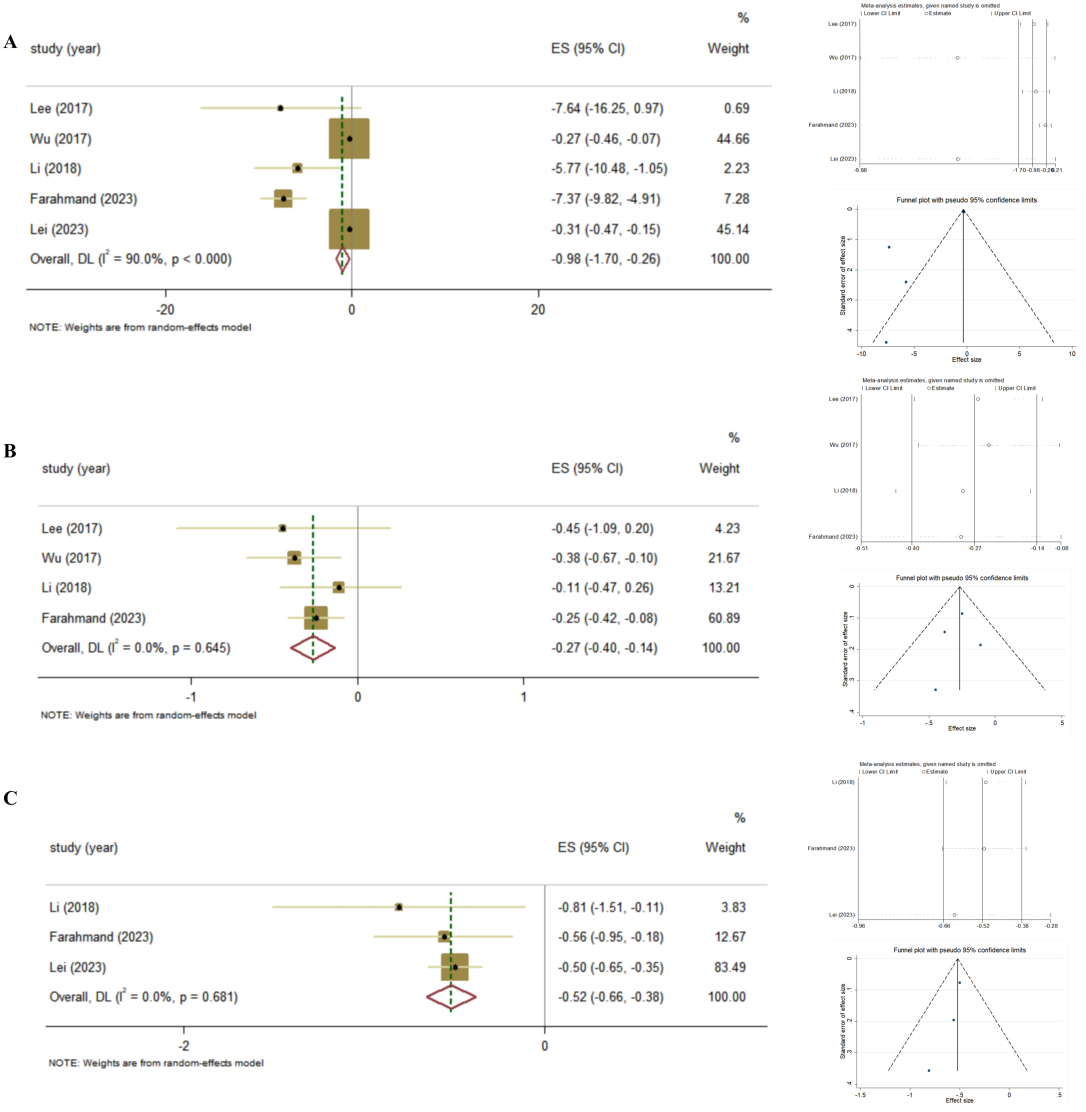
The effect of Vitamin D supplementation on glycemic control in T2DM patients with Vitamin D deficiency according to Meta-analyses of RCT. **(A)** FBG; **(B)** HbA1c; **(C)** HOMA-IR.

Furthermore, we conducted the summary analysis of the impact of Vitamin D supplementation on FBG in patients with Vitamin D deficiency. It was found that, Vitamin D supplementation was more effective on improving FBG in T2DM patients with Vitamin D deficiency, compared to those with adequate Vitamin D status (Pooled ES = −0.98; 95% CI: −1.70, −0.26, *p* = 0.008; I^2^ = 90.0%, *P*-heterogeneity <0.001), and there was no publication bias (Egger’s test, *p* = 0.063; Figure 5A).

#### 3.4.3 Vitamin D supplementation and HbA1c in T2DM patients

The association between Vitamin D supplementation and HbA1c was examined in nine Meta-analyses of RCT, the findings revealed the significant effect (Pooled ES = −0.11; 95% CI: −0.20, −0.02, *p* = 0.02; Figures 4D–F). The level of heterogeneity was slightly higher (I^2^ = 58.2%, *P*-heterogeneity = 0.004). Then, we removed each study separately, and the heterogeneity remained significant. Furthermore, we conducted a sensitivity analysis and the results showed that although the four studies (Lee, 2017, Wu, 2017, Hu, 2019, Farahmand, 2023) influenced the direction of the merged results, heterogeneity still existed after adjustment. Finally, no significant publication bias was observed in funnel plot and Egger’s test (*p* = 0.665).

Also, we conducted the summary analysis of the impact of Vitamin D supplementation on HbA1c in T2DM patients with Vitamin D deficiency. It was found that Vitamin D supplementation had a stronger effect on improving HbA1c in T2DM patients with Vitamin D deficiency (Pooled ES = −0.27; 95% CI: −0.40, −0.14, *p* < 0.001; I^2^ = 0.0%, *P*-heterogeneity = 0.645), and there was no publication bias (Egger’s test, *p* = 0.757; Figure 5B).

#### 3.4.4 Vitamin D supplementation and insulin in T2DM patients

**Figure 6:**
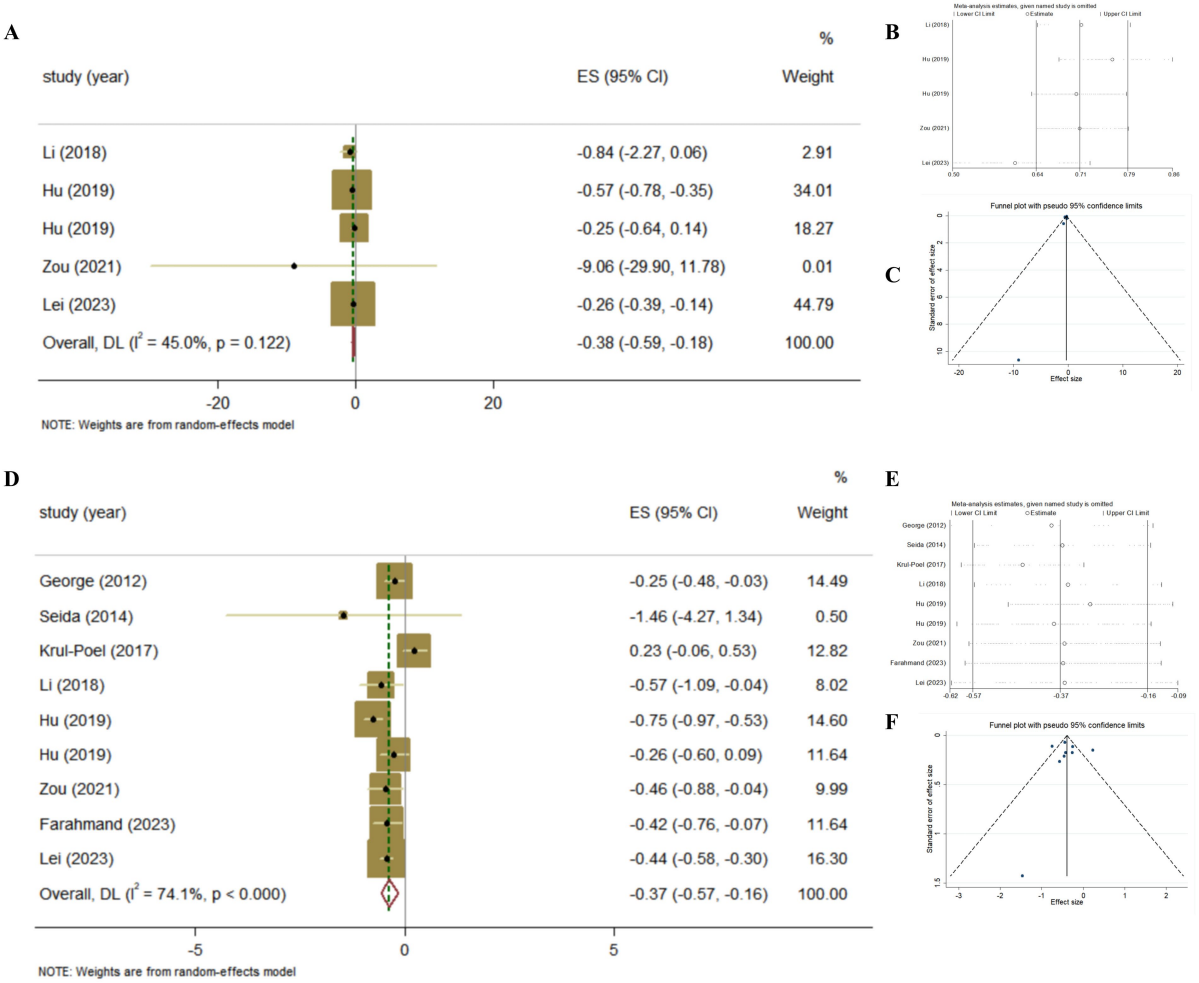
The effect of Vitamin D supplementation on islet function in T2DM patients according to Meta-analyses of RCT. **(A)** Forest plot on insulin; **(B)** Sensitivity analysis on insulin; **(C)** Funnel plot on insulin; **(D)** Forest plot on HOMA-IR; **(E)** Sensitivity analysis on HOMA-IR; **(F)** Funnel plot on HOMA-IR.

The association between Vitamin D supplementation and the level of insulin was examined in 4 Meta-analyses of RCT. The results revealed the significant effect by random-effects model (Pooled ES = −0.38; 95% CI: −0.59, −0.18, *p* < 0.001; Figures 6A–C). The level of heterogeneity was lower (I^2^ = 45.0%, *P*-heterogeneity = 0.122), and the sensitivity analysis and the results showed that there was no significant change in ES and 95% CI, the results of the Meta-analyses were relatively robust. Finally, no significant publication bias was observed by funnel plot and Egger’s test (*p* = 0.367).

#### 3.4.5 Vitamin D supplementation and HOMA-IR in T2DM patients

The association between Vitamin D supplementation and HOMA-IR was examined in 8 Meta-analyses of RCT and the results found the significant effect (Pooled ES = −0.37; 95% CI: −0.57, −0.16, *p* < 0.001; Figures 6D–E). The level of heterogeneity was high (I^2^ = 74.1%, *P*-heterogeneity <0.001). Subsequently, we removed each study, and the heterogeneity remained significant. Finally, we conducted a sensitivity analysis and the results showed that there was no significant change in ES and 95% CI, indicating that the results of the Meta-analyses were relatively robust. No significant publication bias was observed in funnel plot and Egger’s test (*p* = 0.884).

Then, we conducted further analysis of Vitamin D supplementation on HOMA-IR in T2DM with Vitamin D deficiency. As a result, it was found that Vitamin D supplementation was more effective on improving HOMA-IR in T2DM patients with Vitamin D deficiency (Pooled ES = −0.52; 95% CI: −0.66, −0.38, *p* < 0.001; I^2^ = 0.0%, *P*-heterogeneity = 0.681), and there was no publication bias (Egger’s test, *p* = 0.212; Figure 5C).

#### 3.4.6 The quality of evidence on the effect of vitamin D supplementation on glycemic control

We evaluated the quality of evidence on the effect of Vitamin D supplementation on glycemic control (FBG, HbA1c, insulin and HOMA-IR) using the GRADE system, which is currently recognized as the quality of evidence system. It was found that, except for insulin which was evaluated as moderate, the quality of evidence of other outcomes were evaluated as low or very low (Table 3).

**Table 3:** The quality of evidence assessment using the GRADE approach.

Outcome	Risk of bias^a^	Inconsistency^b^	Indirectness^c^	Imprecision^d^	Publicationbias^e^	Quality of evidence^f^
FBG	Serious	Serious	Not serious	Not serious	Serious	Very low
HbA1c	Serious	Serious	Not serious	Not serious	Not serious	Low
Insulin	Serious	Not serious	Not serious	Not serious	Not serious	Moderate
HOMA-IR	Serious	Serious	Not serious	Not serious	Not serious	Low

a Risk of bias based on the AMSTAR results, less than one-third (not serious), one-third to two-thirds (serious), more than two-thirds (very serious).

b Downgraded if there was a substantial unexplained heterogeneity (I^2^ > 50%, *p* < 0.10) that was unexplained by meta-regression or subgroup analyses.

c Downgraded if there were factors present relating to the participants, interventions, or outcomes that limited the generalizability of the results.

d Downgraded if the 95% confidence interval (95% CI) exceeds half of the included Meta-analyses.

e Downgraded if there was an evidence of publication bias using funnel plot.

f Since all included studies were Meta-analyses of randomized clinical trials, the certainty of the evidence was graded as high for all outcomes by default and then downgraded based on prespecified criteria. Quality was graded as high, moderate, low, very low.

FBG, fasting blood glucose; HbA1c, glycosylated hemoglobin, type A1C; HOMA-IR, homeostatic model assessment of insulin resistance.

## 4 Discussion

Up to now, there have been many studies on the prevention and improvement of T2DM with Vitamin D. But the inconsistent conclusions, including Meta-analysis of cohort studies or RCTs, puzzle researchers. Therefore, in order to obtain a clearer conclusion, we conducted this umbrella review to answer the benefits of Vitamin D on T2DM onset and development. As we know, this study is the first umbrella review to summarize existing evidence regarding Vitamin D level and the risk of T2DM, the prevention and improvement effect of Vitamin D supplementation on T2DM.

In order to clarify the prevention of Vitamin D on T2DM, we collected and summarized six Meta-analyses for cohort studies, involving a total of 655,231 participants from 71 studies. It is interesting to note that, although there have been inconsistent conclusions in published cohort studies on the increased risk of T2DM due to Vitamin D deficiency. The conclusions drawn from Meta-analysis are mostly meaningful after summarizing studies with inconsistent conclusions. As we included in the six Meta-analyses, only one study concluded that Vitamin D deficiency was not associated with the onset of T2DM. However, the re-aggregation of Meta-analysis strongly confirmed the positive correlation between lower Vitamin D levels and T2DM and demonstrated that lower serum 25(OH)D level was risk factor for T2DM. After sensitivity analysis, this conclusion is still robust.

Besides the cohort studies on the relationship between Vitamin D levels and the onset of T2DM, we also focused on the prevention of Vitamin D supplementation on T2DM. Indisputably, as diagnostic criteria and main pathological features of T2DM, FBG and HbA1c are represented the short-term and long-term glucose homeostasis, respectively (23). Besides, in order to resist the hyperglycemia, the maintenance of islet function is indispensable (24). Thus, we also picked insulin level and HOMA-IR to judge whether Vitamin D led to the occurrence of T2DM. More importantly, multiple mechanism studies claim that the protective action of Vitamin D on T2DM is mainly achieved through the above indicators (25). However, in our systematic review of Vitamin D supplementation for the prevention of T2DM, we found that Vitamin supplementation showed null effects on FBG, HOMA-IR, and HbA1c in non-T2DM population. Similarly, the another Meta-analyses included 9 RCTs (43,559 participants) studying Vitamin D supplementation in the prevention of T2DM and found that the supplementation of Vitamin D failed to prevent T2DM in the general population, the RR for Vitamin D compared with placebo was 0.96 (95% CI, 0.90, 1.03) (26).

However, Vitamin D supplementation seems to have an effect on prediabetes. The Vitamin D and type 2 diabetes (D2d) trial is funded by the National Institutes of Health (NIH) to test whether Vitamin D supplementation reduce the risk of T2DM in prediabetes with an average follow-up time of 2.5 years. Although the D2d trial, published in the New England Journal of Medicine in 2019, showed an increase in average serum 25(OH)D levels in the Vitamin D intervention group (daily supplementation with 100 μg of Vitamin D_3_), the HR was 0.88 (0.75–1.04) (27). While, the D2d trial published in the Diabetes Care in 2020 showed that the HR among participants treated with Vitamin D who maintained 25(OH)D levels of 100–124 and ≥ 125 nmol/L were 0.48 (0.29–0.80) and 0.29 (0.17–0.50), respectively, compared with those who maintained a level of 50–74 nmol/L (28). More importantly, the latest Endocrine Society Clinical Practice Guide on Vitamin D for the prevention of diseases recommends, for adults with high-risk prediabetes, empiric Vitamin D supplementation to reduce the risk of progression to diabetes is suggested, and the anticipated desirable effects are likely moderate. Besides, the panel not only reasoned that there are likely cost savings with using Vitamin D for diabetes prevention, but also believed that the benefits of Vitamin D supplementation may preferentially accrue to those at highest risk for Vitamin D deficiency (29).

Furthermore, our focus shifts to T2DM patients, and we hope to clarify the improvement effect of Vitamin D supplementation on glycemic control in T2DM patients. We collected and summarized 10 Meta-analyses for RCTs, involving a total of 53,198 participants from 80 RCTs. The umbrella review about RCTs verified Vitamin D supplementation remarkable improved glucose homeostasis and islet function. For indicators of glucose homeostasis, all studies tested FBG and nine studies tested HbA1c. Although less than half of the studies have shown that Vitamin D supplementation improved glucose homeostasis, the final pooled effect size was still meaningful. However, the interpretation of FBG need to be caution. Because the study of Farahmand, 2023 in the results was the source of heterogeneity, while the results become meaningless when removed this study. However, upon reexamination, it was found that the vast majority of RCT trials included in this study were also included in other Meta-analyses. In addition, for indicators of insulin homeostasis, although only some studies detected insulin and HOMA-IR, most of the results showed meaningful results. Subsequently, the quality of evidence on the effect of Vitamin D supplementation on glycemic control (FBG, HbA1c, insulin and HOMA-IR) was evaluated using the GRADE system. It was also found that, except for insulin which was evaluated as moderate, the quality of evidence of other outcomes were evaluated as low or very low.

From the pooled results and the quality of evidence, the improvement effect of Vitamin D on T2DM is more concentrated on the islet function. We further attempt to provide the mechanistic explanation. The notion that Vitamin D supplementation may ameliorate glucose homeostasis and islet function, especially the levels of insulin, in T2DM patients is physiologically feasible. First, Vitamin D affects the production and secretion of insulin. All of the Vitamin D functional components, including the Vitamin D receptor (VDR), hydroxylase Cytochrome P450 27B1 (CYP27B1), and Vitamin D-binding protein, have been identified in the pancreatic islet cells (5). Due to the presence of the Vitamin D response element in the promoter region of the insulin gene, the genomic pathway induced by 1,25(OH)_2_D_3_ in pancreatic *β*-cells, which express both VDR and CYP27B1, increases the synthesis and secretion of insulin. Relevantly, in VDR-Knockdown mice, beta cells have difficulty converting proinsulin into insulin (7). Moreover, evidence suggests the link between insulin signaling and Vitamin D-mediated insulin sensitivity. In addition to insulin, 1,25(OH)_2_D_3_ also facilitates the transcriptional activation of the insulin receptor gene, which increases the number of insulin receptors on the surface of cells that respond to insulin (6, 7). Therefore, from the perspective of mechanism, Vitamin D status not only affects the occurrence of T2DM, but also affects the development of prediabetes and diabetes patients.

However, numerous recent research found that the status of Vitamin D affects the improvement of Vitamin D supplementation on T2DM (4, 30–35). In fact, currently negative findings of RCTs on the effect of supplementation of Vitamin D on incidence or outcomes of T2DM are affected by relevant methodological design bias. Since some studies did not detect the Vitamin D status of participants, or very few of them (far less than half) were severely Vitamin D deficient (36). This may represent an important limitation because Vitamin D supplementation is not likely to be effective for those subjects with sufficient Vitamin D. As a threshold nutrient, giving more of the needed dose of Vitamin D (pharmacological vs. physiological approach) may not necessarily lead to beneficial effects. Therefore, based on the Meta-analyses of included Vitamin D supplementation, we conducted an in-depth analysis of the improvement effect of Vitamin D supplementation in T2DM patients with Vitamin D deficiency. The results showed that for T2DM patients with Vitamin D deficiency, Vitamin D supplementation resulted in more significant improvements in glucose control and islet function.

Noteworthy, both T2DM and hypovitaminosis D may be considered modern pandemics. While, the prevalence of T2DM with Vitamin D deficiency has become increasingly severe. The Meta-analyses unpacking the Vitamin D deficiency rate in T2DM patients showed that after inclusion in 51 studies, the pooled prevalence of Vitamin D deficiency in T2DM patients was 64.2% (95% CI: 60.6, 67.8%). Moreover, the study also revealed that the incidence of Vitamin D deficiency in T2DM patients was increasing each year, the prevalence of Vitamin D deficiency from 2018 to the recent 65.0% was higher than the prevalence before the year 2018 (63.5%) (36). Therefore, future RCTs should focus on Vitamin D deficient subjects, and intervened them with Vitamin D supplementation to achieve and maintain sufficient Vitamin D levels.

There were few limitations that must be noted as well. First, the heterogeneity reported in partial analyses was relatively high. The subgroup analysis was regretfully conducted due to the scattered characteristics in including Meta-analyses. However, no significant changes were found in the sensitivity analysis. Publication bias was only found in the analysis of FBG, and was not found for others. Therefore, we speculate that the higher heterogeneity could possibly be the results of the age group or the duration of diabetes and the complexity of the disease. Moreover, not all included Meta-analyses considered the effect of environmental factors such as sunlight, altitude, or diet on Vitamin D status. Additionally, not all trials examined or reported a participant’s baseline serum Vitamin D level, since low serum Vitamin D levels impact both the therapy with Vitamin D supplementation and the symptoms of T2DM. Eventually, RCTs comparing calcitriol, ergocalciferol, cholecalciferol, and alfacalcidol against placebo or no treatment were eventually included in the Meta-analyses; several of these studies did not even specify the type of Vitamin D supplementation that was being taken. As such, even with our greatest efforts and performing the procedure, we were unable to distinguish the effects of the different Vitamin D supplementation.

Notwithstanding these limitations, the current umbrella Meta-analyses have several strengths. The most noteworthy advantage of this analysis is the inclusion of several observational studies and RCTs of high and/or moderate-quality based on the AMSTAR2 questionnaire. Another advantage was evaluating the quality of evidence between the effect of Vitamin D supplementation on glycemic control using the GRADE system, which is currently recognized as the quality of evidence system. Although only insulin was classified as moderate in the evaluation, our results still provide appropriate recommendations on Vitamin D supplementation for T2DM patients, especially for those with Vitamin D deficiency. Meaningfully, the present study supports that Vitamin D supplementation could be considered a beneficial adjuvant therapy on glycemic control for T2DM patients, especially with Vitamin D deficiency.

## 5 Conclusion

The present umbrella Meta-analyses demonstrates that lower serum Vitamin D levels accelerate the onset of T2DM and confirms that Vitamin D supplementation fails to prevent T2DM. However, in T2DM patients, the potential benefits of Vitamin D supplementation on ameliorating glucose homeostasis and islet function has been verified, especially in those with Vitamin D deficiency.

## Footnotes

^1^ www.crd.york.ac.uk/PROSPERO
